# Yoga studio websites: are they an accurate first glance at the studio’s mission, values, and resources?

**DOI:** 10.1186/s12889-023-16560-4

**Published:** 2023-08-25

**Authors:** Anna Dysart, Jake Barnett, Samantha M. Harden

**Affiliations:** 1 Human Nutrition, Foods, and Exercise, Virginia Tech, Blacksburg, VA USA; 2 Virginia Tech, Neuroscience, Blacksburg, VA USA

**Keywords:** Physical activity, Health promotion, Health communication

## Abstract

**Background:**

Yoga, as an ancient and modern practice, increases physical, mental, emotional, spiritual, and social health. Yoga studio websites serve as a dissemination channel for studios to express their offerings, whom they employ, and whom they seek as clientele. Public health workers, physicians, researchers, and clinicians, can refer to existing studios to increase health among their patients or clients. The degree to which these websites can provide relevant information to these various stakeholder groups has yet to be defined.

**Methods:**

A pragmatic, sequential mixed-methods study was employed with quantitative data extraction, summarized as means and proportions, to score the studio websites (*N* = 28), and semi-structured interviews (*n* = 6) analyzed using the rigorous and accelerated data reduction (RADaR) technique, to confirm website content and staff intention. To explore urban and rural characteristics, yoga studios in southwest Virginia and Los Angeles were selected for inclusion.

**Results:**

Overall, community-based yoga studios websites included information on the type, duration, cost, and COVID mitigation strategies. The most common class duration was 60 min. Rural Southwest Virginia studios offered 8.5 classes per week whereas those in urban Los Angeles offered 24.2 classes per week. All studios used iconography and images to invite racial, ethnic, age, and body type and ability diversity. While studios in both areas specified that there were 200- and 500-hour registered yoga teachers, many of the instructor biographies did not include information on their training. Although only preliminary, the interviews (*n* = 6) confirmed that the websites generally represented the feel, intention, and offerings of the studio and that the primary purpose of the studio was to build relationships and ensure people felt comfortable in the space.

**Conclusion:**

Website information was related to studio offerings and values; however, discussion with management or visiting the studio may provide a richer picture of the yoga practices offered in the space. Further suggestions for website content are provided.

**Supplementary Information:**

The online version contains supplementary material available at 10.1186/s12889-023-16560-4.

## Background

The practice of yoga in the United States is gaining in popularity, and with over 6,000 yoga studios in the United States, those wanting to practice yoga have a wide variety of studios to choose from [1]. Research has focused on yoga and emotional regulation, i.e. stress reduction, [2–4] worry [3] and anxiety [3]. Other research has focused on how yoga might be beneficial for a range of different populations, including older adults [5–7] and university communities [2] in both physical aspects (fall prevention, [2, 6, 7] aerobic activity, [5] muscle strengthening and flexibility [5]) and emotional regulation [2–4]. The integration of information about qualifications and offerings for specific populations on yoga studio websites could serve as a dissemination channel for yoga practice promotion by health educators and professionals. This information may be especially useful for professionals who refer to existing community entities and who are looking for safe, effective mind-body offerings after the COVID-19 outbreak.

**Fig. 1:**
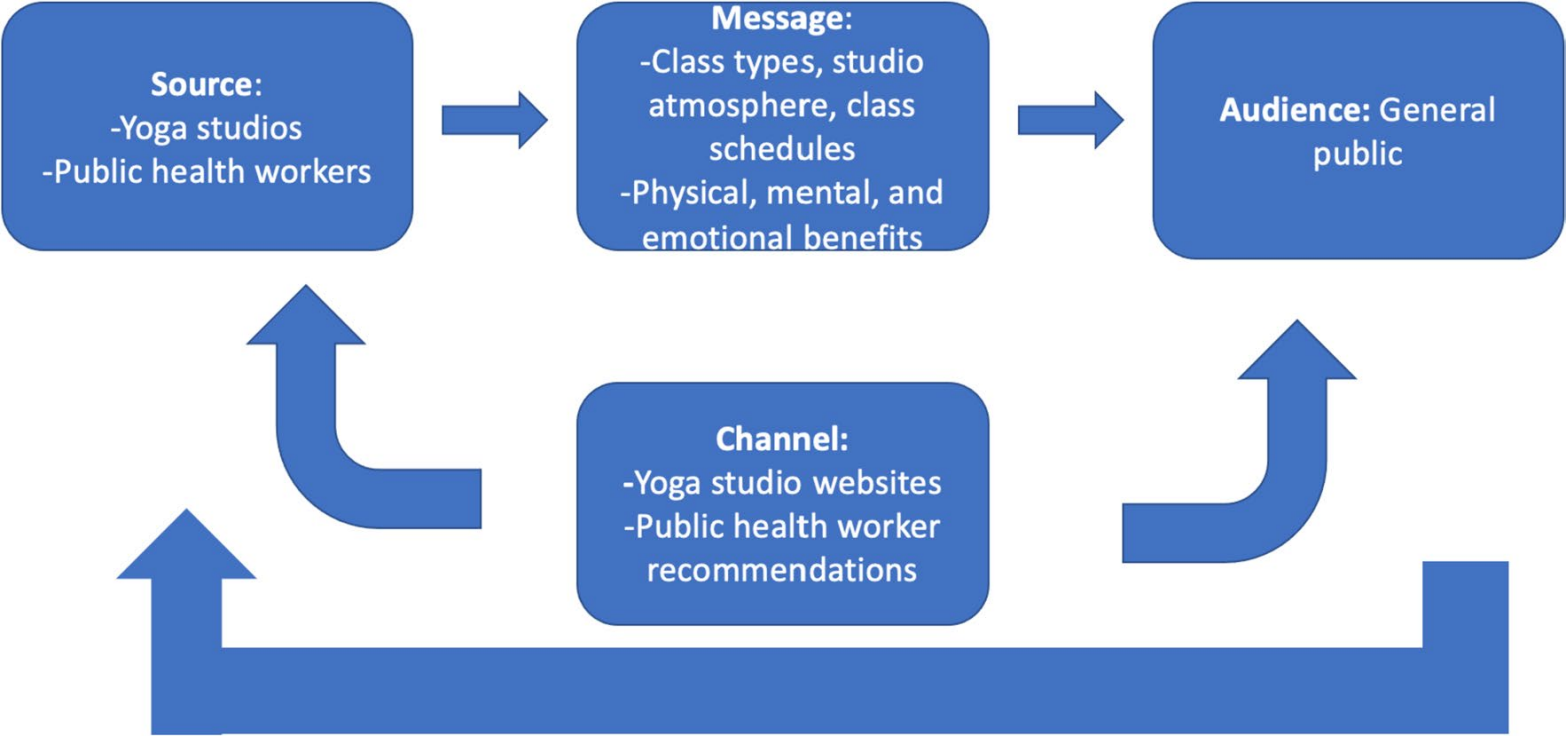
Modified dissemination model from Brownson, et al. first published in J Public Health Manag Pract. 2018

Although yoga in the U.S. represents a 16 billion dollar industry, [1] research acknowledging the yoga studio and its representatives (owners, managers, teachers) as an integral part of the broader yoga community and their influences on the participants physical and emotional health is nascent. Yoga studios follow a wide range of teaching philosophies and offer a multitude of different class types, experiences, and lengths [1]. People wishing to begin a yoga practice may be unsure of the terminology, etiquette, or typical cost involved with yoga classes and one of the top ways they get more information about yoga and physical activity behavior changes is through the internet [1, 8]. Therefore, as the studio website may be the first interaction between industry professionals and their clients, it is important for yoga studio websites to clearly convey the studio’s unique yoga philosophy and teaching/class structure to assist beginners in choosing the right studio for them. The studio website serves as a channel within a dissemination strategy—both the messaging and the intended audience matters when trying to draw in attendees and increase physical activity (see Fig. 1) [8, 9]. Having websites that meet readability and suitability standards [8] as well as utilizing emotion and demonstrative usefulness to the audience [9] may increase participant draw into the studio.

Furthermore, during the COVID-19 pandemic, many yoga studios were forced to close per health department regulations. However, new and old yoga participants continued to turn to the breathing and mindfulness inherent in yoga practice as a way to combat the physical and emotional tolls of the pandemic [10–12]. Multiple editorials touted the immune system prompting effects of yoga as a prophylactic measure against COVID-19, [10–12] while others outlined the preliminary results of using virtual yoga to combat the stress during pandemic times [10].

Although classes taught in a yoga studio may not meet formal definitions of evidence-based interventions, they align with the notion that if we want more evidence-based evidence, we need more practice-based evidence. Typical yoga classes offered in community settings include breathwork, meditation, movement, and building a sense of community—all of which are protective of mental and physical health deterioration. However, there has been limited participatory work linking the yoga industry with empirical practices.

Therefore, community-based educational systems that serve as prevention medicine [13, 14] have a prime opportunity to refer to yoga studios. These prevention system health educators are frequently on the front lines of dissemination and the spread of knowledge gathered through research [15]. It is important for health educators and professionals, to have a baseline for what the images and language on yoga studio websites reflect for the potential physical and emotional health benefits of attendees.

The purpose of this study was to extract data related to the information, imagery and language on yoga studio websites and inform the development of a yoga studio assessment. These data were coded by registered yoga teachers and non-yoga participants to have a variety of perspectives. Assessing a key dissemination channel can inform community-based health educators, in their referral process. Due to the unique way public health workers connect with a wide array of the population as a preventative measure, [13, 14] it is important for them to be able to appropriately refer people to yoga studios and yoga classes. This may ultimately increase the proportion of Americans accessing a yoga practice for mind, body, and spiritual health.

## Methods

### Overview

Yoga studios were reviewed using a sequential mixed methods design [16]. First using a data extraction technique looking at the yoga studio websites, followed by qualitative investigation utilizing a semi-structured interview technique. Studios were chosen from two regions, the New River Valley in Virginia and Los Angeles, California. These regions were chosen to explore potential differences between a more rural area with a population of approximately 180,000 [17] and a metropolitan area with over 10 million people [18].

### Studio selection

Studios were found online through searches on the Yoga Alliance website and top search engines. Yoga Alliance is non-profit organization based in the United States that supports and advocates for standards for studios and teachers [19]. Studios/teachers within an 85 mile radius of New River Valley, Virginia, and a 15-mile radius of Downtown Los Angeles, California with a website linked to the Yoga Alliance website were recorded. Search engines were used to expand the pool of studios examined, as not all studios are registered with Yoga Alliance. Due to the limited number of studios in the New River Valley region versus the Los Angeles region, studios were limited to 15 in each region (the number of studios found in the New River Valley).

### Studio coding

Data extraction variables were developed from input of the coders, who had varying familiarity with yoga. This was important in order to reflect (as best able) the types of people who may be visiting the yoga studio websites for information. The coders were an undergraduate research assistant who was not a yoga practitioner; a graduate research assistant who was a 200-hour registered yoga teacher (RYT); and a behavioral psychologist who was a 500-RYT. The undergraduate and graduate research assistant were trained in qualitative research methods by the behavioral psychologist, who has years of experience in qualitative research and research publications utilizing similar coding methods. Coding categories were developed to encompass details such as diversity, studio culture, and attitudes that a health professional might want to know in order to recommend a yoga studio to clients or participants [13, 20, 21]. The coding strategy was developed off of similar work done with the dissemination of physical activity guidelines information on open access websites [22]. Coding of websites took place during the COVID-19 pandemic when studios in both regions were unable to be open. This was accounted for as one of the categories and availability of online classes was also determined. See Additional file 1: Appendix 1 for full list of data extraction variables. Coders also had access to the coding companion sheet (Additional file 1: Appendix 2), which explored the categories and answers in more depth to promote consistency across the coders. All coders extracted data from two eligible studio websites from the Los Angeles region and two from the New River Valley region to establish inter-rater reliability. Inter-rater reliability was determined to be high (> 90%). Any differences in coding were resolved by the third coder.

Data obtained by the coding process was then evaluated to determine trends across the studios in each region and overall. Categories that were frequently not found on the websites were used as the basis for development of the interview questions.

### Semi-structured interviews

Semi-structured interviews of the studios were completed by coder 1. This was determined to be non-human subject research by the university IRB. Studios were first contacted through the phone number listed on their website. This method received the best results from studios in the New River Valley region. No studios in Los Angeles responded when contact was attempted via phone call. Due to lack of response, emails and then text messages were sent to the studios that were not reached by phone utilizing the information found on the studio website. If there were no contact methods available, the studio was not contacted for an interview.

Interviews were recorded by the interviewer with permission from the interviewee. Interviews followed a semi-structured guide. Attempts were made for the interviewee to be a studio manager or owner in order for more knowledgeable answers. Following each interview, the interviewer transcribed the interview verbatim so that accurate analysis of the interviews could be completed.

### Interview analysis

The transcripts of the semi-structured interviews were analyzed using a rapid analysis technique to derive the overarching themes for all the interviews [23]. First, each interview was separated into the main themes that appeared in the interview. This was the broadest table, which was then reduced further following the rigorous and accelerated data reduction (RADaR) technique into smaller tables that narrowed in on the themes that pervaded all the interviews [23]. See Additional file 1: Appendix 3.

## Results

In total 28 yoga studio websites were coded, 15 in Los Angeles (LA), California and 13 in the New River Valley region of Virginia (VA).

### Quantitative results- website disparities and similarities

LA had more studios registered with the Yoga Alliance (40%), as well as more studio chains when compared to the VA region. Both VA and LA studios had COVID-19 messages; however, a greater proportion (73.3%) of the LA studios provided a COVID context, such as what they were doing to mitigate the spread of COVID, to their website. The LA studios had a higher average of 58.8 Google reviews and 135.9 Yelp reviews, while the VA studios had an average of 1.2 Google reviews and 5.5 Yelp reviews. LA studios earned lower star ratings out of 5 on both Google (4.3) and Yelp (4.4), while the VA studios received an average of 4.6 on Yelp and 4.98 on Google. The LA studios had higher ethnicity diversity, age, and gender diversity visible on their websites, while the VA studios had higher body type and body ability diversity.

More studios in LA offered classes early morning classes between 5:00 am and 8:00 am. Both LA and VA studios offered more morning and evening classes than any other time. LA also offered more night classes after 7:00 pm until midnight. The average number of classes offered in the LA studios was 24.2, while the VA classes offered 8.5 classes a week. However, this average for VA studios was calculated from schedules of available class schedules before the COVID-19 pandemic. On the available schedule during the pandemic, the VA studios offered an increased number of classes per week at 9.375 per week.

The average monthly cost of membership was higher for the LA studios and the LA studios also charged more for a single class, averaging $20.46 per class versus $14.09 in the VA studios. The max price of private classes was higher in LA at $125; however, both locations offered a broad range of prices depending on the teacher. Both LA and VA studios’ most frequent class length was 60 min. The longest class length was 150 min for the LA studios, while the VA studio’s longest class time was 120 min. For both LA and VA, the most frequent class style was “Flow”. A larger percentage (30.7%) of the VA studios offered a pay-scale or donation system for classes.

While both VA and LA locations had studios that used social media, a larger proportion of the LA studios used Facebook, Twitter, and Instagram. The LA studios also had more newsletters and blogs for attendees. A small proportion of the studios in both locations (1 in VA and 3 in LA) offered competitions and prizes for attendees.

The mean number of teachers was higher for the LA studios at 9.9 teachers per studio versus VA studios that had a mean of 7.83 teachers per studio, though the number of 200RYT instructors was higher in the VA studios. Both regions had an equal number of 500RYT (5 per studio on average), and LA had a higher number of instructors with other certifications or none listed. The LA studios were also more likely to offer teacher training. The LA studios also listed more benefits of exercise, meditation, and nutrition recommendations on their websites.

### Phone interviews

The six phone interviews were 14.97 (± 9.01) minutes in duration. Thematic patterns emerged from the interviews provided by studio staff. Notable differences were present between studios that had been open for a more extended period of time with the more modern studios. These differences were present regardless of the location, and were concerned with the style of yoga practice offered. Two-thirds of the studios tracked popularity of yoga classes through attendance, also citing the teacher as one of the main reasons a class is popular. Morning and evening were the most popular times for classes to be held across both regions. All the studios thought the relationships they have with their participants were very important in making attendees feel welcome. See Table 1 for more information on themes.

**Table 1:** Qualitative themes resulting from analysis of semi-structured interviews

**Studio**	**Studio Open Date**	**Studio Goal**	**Website Reflection**	**Social Media Recruitment**	**Popularity Tracking**	**What Makes a class Popular**	**Most Popular Class Time**
**Breathing Space**	1999	End Suffering	Pleased	None	No Tracking	--	Morning/Evening
**Just Breathe**	2008	Spread yoga to new people	Needs Tweaks	--	No Tracking	Individ-ualized Attention	Morning
**Studio 221**	2017	Spread yoga to new people	Needs Tweaks	Facebook, Instagram	Attendance	Teacher and Timing	Morning
**Vita-Zen**	2010	Spread yoga to new people	Pleased	Facebook, Instagram, Twitter	Attendance	Teacher, Class Type	Afternoon
**Uttarra**	--	Spread yoga to new people	Pleased	Facebook, Instagram	Attendance	Time, Teacher	Morning
**Yoga Circle Downtown**	1999	Spread yoga to new people	Needs Tweaks	Not important	Attendance	Time, Teacher	Evening
	**What Class Style is Most Popular**	**How do you make attendees feel welcome**	**Diversity-Staff**	**Diversity-Students**	**8 Limbs and/or Sanskrit**	**Minimum Teacher Qualification**	
**Just Breathe**	--	Relationship	Age	--	Both	--	
**Studio 221**	Iyengar	Relationship	--	age	Yes	200 RYT	
**Vita-Zen**	Adaptive Gentle	Relationship	None	Body Ability	Only Sanskrit	200 RYT	
**Uttarra**	Ageless Yoga	Relationship	None	Body Size, age, race	Both	200 RYT	
**Yoga Circle Downtown**	Relationship	Diverse	Diverse	Both	Unsure, No minimum	--	
**Breathing Space**	Relationship	Age, Ethnicity, Gender	--	Both	Experience/hard certification	--	

## Discussion

Through this mixed-methods work we were able to compare studios from different regions of the country at a very transitional time for yoga studios. While many of these comparisons are unsurprising due, primarily, to the geographic, demographic, and financial differences between LA and VA, they lay the foundation for what some of the previously unidentified yoga industry norms are from an empirical perspective. There were some key differences between studios even in the same region that arose when examining the websites and the ‘why’ of yoga practice, though those differences were less apparent in the studios’ handling of the COVID-19 pandemic. Overall, studio websites reflected the mission and offerings of the studios (as confirmed via qualitative data), and we provide suggestions for studio websites to facilitate potential partnership with health professionals.

Many of the studios noted the number of training hours their instructors had completed while some had no information about the specific training or credentials of their instructors. Therefore, details on where, how, and what the instructor was capable of (i.e., does the instructor have a training for yoga for cancer patients? Youth? Older adults? Pregnant persons?). Without this information, a referral process may be limited. Notably, aside from yoga therapy specifically, yoga instructors in the United States “pay to play” through registration with Yoga Alliance after completing training with a Yoga Alliance Registered School. Although Yoga Alliance is not a governing body nor is there a comprehension exam (credentialing), previous intervention studies have valued Yoga Alliance registration, relying on instructors having a minimum of a 200 h RYT certification [7]. Therefore, studio websites may want to include this information for potential partnership with researchers, public health workers, and other referral mechanisms.

Via the interviews with studio representatives, and in alignment with the mission of spiritual growth, the comprehension of the spiritual and transcendent benefits of yoga emerged as a primary criterion for hiring teachers for these studios. The owners of these studios valued experience with the 8 limbs and knowledge of ‘true yoga’ in a potential teacher. Knowledge of poses and continued training has been highlighted as important both in utilizing yoga instructors in research studies and studies on the yoga industry [1, 6]. Interestingly, the yoga focused locations spoke primarily of ending suffering or offering a reprieve from life for the attendees, while the ‘yoga as exercise’ studios primarily focused on introducing yoga to new people as a primary goal. Finally, studio representatives pointed to the desire to “build relationships and ensure people felt comfortable in the space,” which may be backed through employee manuals, website statements, etc. for more transparency on how this is accomplished.

The use of websites and social media by yoga studios has evolved, with many studios commenting that they use social media and the web. Most studios were pleased with their websites. Others stated that it needed a few adjustments after the COVID-19 pandemic stating, “it doesn’t reflect because we’re a virtual studio. If we were still brick and mortar, it would be wonderful.” Tele-yoga, or yoga being done virtually instead of in-person, has been discussed as a reasonably accepted response to the COVID-19 pandemic necessitating the move of classes online [10]. Additionally, the VA studios were able to expand the number of classes offered during the pandemic, and therefore were able to reach a wider audience. Even prior to the pandemic, yoga students who chose to practice outside of structured classes frequently chose to use instructional materials such as pre-recorded classes [24]. Continued changes to the way yoga classes are presented both live online and pre-recorded may happen as the world adjusts to COVID-19 being endemic.

Interestingly, several studios mentioned that during the pandemic, the reach of their studio has increased. The COVID-19 pandemic has put an increased premium on efficient online interfaces, and small businesses like yoga studios had to adapt their business model. The online presence of the studio was prioritized and became the primary location for conducting many classes during the pandemic. As the pandemic caused an increase in reported depression, anxiety, and stress, [25] many yoga practitioners continued to turn to their practice as a way to mitigate the effects of the pandemic [10]. Those who promote health and wellness as their work, like yoga studios, face a great deal of adaptability challenges, and must continue to evaluate their use of technology.

Studios also stated that streamed classes online increased the diversity of attendees. In addition to increased diversity, class times that were less popular saw higher attendance levels. One studio owner also mentioned that the studio was also experiencing more participation from individuals in different time zones and even different countries. Allowing participants to practice in the comfort and safety of their own homes may promote a willingness to try new activities that they would not have practiced before, with individuals having reported in the past that barriers to participating in yoga included lack of childcare and inconvenient studio locations [24].

While this work focuses on the degree to which yoga studio websites were representative of their mission, values, and resources, the generalizability and scientific implications of this work are twofold. First, there is generalizability in the website evaluation process. That is, similar methodologies could be employed to rate and review other industries or offerings (such as personal trainers for individuals with chronic pain). This is especially relevant since care coordinators (individuals who support patients or clients) may review an entity’s website (searching for key factors) to help refer individuals to the practice [9, 26]. Secondly, this advances recommendations for websites, since websites themselves sit at the intersection of being a source, channel, and message (see Fig. 1)—a prime opportunity to apply dissemination science to see “what works” to bridge interventions to their intended audiences.

### Implications for practice

Websites are a key dissemination channel for information related to a yoga studio’s mission and resources. Websites are also a tool for public health workers to link potential yoga practitioners with appropriate offerings and settings. Readily available information includes factors such as time and costs. Other relevant information might be related to accessibility factors (e.g., classes appropriate for cancer survivors or older adults). A more in depth look at the safety precautions may also be beneficial for the studio websites to be more applicable to broader populations. Future work is needed to refine and test a studio audit tool to provide systematic guidance for referrals to existing yoga studios. This may increase the number of Americans who can have access to a safe, equitable, and impactful yoga practice.

## Conclusion

The practice, industry, and empirical findings of yoga in the United States continues to grow. However, the end-users (yoga practitioners), industry (owners, managers, and teachers) and science (interventionists) remain siloed. We know that websites provide on-demand information for people to assess a given entity, in this case, a yoga studio. This information can facilitate the degree to which a public health worker, physician, or other provider (e.g., psychologist) may feel confident referring patients/clients to an existing yoga studio. The yoga industry is looking for ways to share their offerings, increase and maintain clientele. Yoga researchers are looking for ways to scale and sustain evidence-based practices. This study investigated information that the industry has readily provided and shares suggestions to improve linkages between what different end-users may need in order to access the studio’s offerings.

### Supplementary Information

**Additional file 1:** **Appendix 1. **Yoga studio study coding. **Appendix 2.** Yoga studio coding companion sheet - further explanations/definitions. **Appendix 3.** Qualitative themes resulting from analysis of semi-structured interviews.

**Additional file 2.**

## Data Availability

The dataset (Yoga Studio Website Data Extraction sheet) used is attached as a supplementary file.
