# Supplementary material for: Yoga studio websites: are they an accurate first glance at the studio’s mission, values, and resources?
Source: BMC Public Health. 2023 Aug 25;23:1622. doi: 10.1186/s12889-023-16560-4 (PMC10464212; doi:10.1186/s12889-023-16560-4)
Supplement: Supplementary file 1 — Additional file 1: Appendix 1. Yoga studio study coding. Appendix 2. Yoga studio coding companion sheet - further explanations/definitions. Appendix 3. Qualitative themes resulting from analysis of semi-structured interviews. [file 12889_2023_16560_MOESM1_ESM.docx]

Appendix 1

**YOGA STUDIO STUDY CODING**

*All moderators can be given a 999 as well if information was missing

*Leave ‘describe’ cell (in excel doc) blank if answer to previous question was ‘0: no’ (e.g., If website does not mention exercising for health, write ‘0’ under **Exercise for Health** column and leave cell empty under **describe** column… if it does mention exercising for health, you would, write ‘1’ under **Exercise for Health** and ‘yoga is a great exercise to improve health’ under **describe** column)

*Identify any mixes of variables with an ’x’ and then the combination of numbers (If website included a an online schedule and a downloadable schedule, denote this by coding as ‘x12’)

*Identify any exceptions with a new number and then a description (e.g., if coding has ‘0’ or ‘1’, code exceptions as ‘2: [description]’; if coding has ‘0’, ‘1’, ‘2’, or ‘3’, code exceptions as ‘4: [description]’; etc). For example, if **Yoga Style** was something not listed, code as ‘5:surfing yoga’

Reviewer:

1: Anna

2: Jake

3: Samantha

Year Established

Actual year

999: Not reported

Google Reviews

Actual Number of Google Reviews

Google Review Stars

Actual number for star rating

Yelp Reviews

Actual number of Yelp Reviews

Yelp Review Stars

Actual number for star rating

Chain

0: No

1: Yes

Studio Type:

1: gym

2: boutique

Studio Style:

0: No

1: Yes, they specifically mention an overarching style (doesn’t matter what kind)

Describe studio style, i.e. Bikram

Registered with Yoga Alliance?

0: No

1: Yes

2: Other

Describe other

Studio_Awards/Certifications

0: No

1: Yes

2: Other

COVID_Offering classes online?

0: No

1: Yes

2: Unsure

COVID_Message_Copy Directly

999 Not available

Diversity- Race/Ethnicity:

0: No

1: Yes, multiple ethnicities shown in a photo

2: Yes, discussed in text

3: Yes, in both text and photo

Diversity- Age:

0: No

1: Yes, multiple ages shown in a photo

2: Yes, discussed in text

3: Yes, in both text and photo

Diversity- Body type:

0: No

1: Yes, multiple body types shown in a photo

2: Yes, discussed in text

3: Yes, in both text and photo

Diversity- Gender:

0: No

1: Yes, multiple genders shown in a photo

2: Yes, discussed in text

3: Yes, in both text and photo

Early morning classes:

0: No

1: Yes

2: No schedule available

Morning classes:

0: No

1: Yes

2: No schedule available

Afternoon classes:

0: No

1: Yes

2: No schedule available

Evening classes:

0: No

1: Yes

2: No schedule available

Night classes:

0: No

1: Yes

2: No schedule available

Total number of classes, reported by studio

999 not reported

Actual number

Total number of classes, calculated by reviewer

999

Counted by coder

Private_Class: Private classes available/supported

0: No

1: Yes

Private_Class_Cost

999

Actual $

Type of schedule:

1: available online, you do not have to download it to see the full schedule

2: you have to download the schedule

3: call for schedule

4: no schedule available or unsure

Type of classes- yoga style:

Yoga Style_Hatha

0: No

1: Yes

Yoga Style: Power

0:No

1: Yes

Yoga Style: Ashtanga

0:No

1: Yes

Yoga Style: Kundalini

0: No

1: Yes

Yoga Style: Bikram

0:No

1: Yes

Yoga Style: Barkan

0:No

1: Yes

Yoga Style: Hot

0:No

1: Yes

Yoga Style: Aerial

0:No

1: Yes

Yoga Style: Flow- general

0:No

1: Yes

Yoga Style: Yoga combined with another class (yogalites (yoga+pilates), yoga kickboxing, etc)

0:No

1: Yes

Yoga Style: Animal yoga (goat yoga, pig yoga, dog yoga, etc)

0:No

1: Yes

Yoga Style: Prenatal

0:No

1: Yes

Yoga Style: Older adult

0:No

1: Yes

Yoga Style: Other

0:No

1: Yes

Describe other

8 Limbs

0: No

1: Yes, they mention “Yoga Limbs” and discuss or label only one limb

2: Yes, they mention “Yoga Limbs” and discuss or label 2-7 limbs of yoga

3: Yes, they mention all “8 limbs of yoga”

Temperatures Reported

0: no

1: yes

Room_Temperature_62-68

0: no

1: yes

Room_Temperature_68.1-88

0: no

1: yes

Room_Temperature_88.1-100

0: no

1: yes

Room_Temperature_>100

0: no

1: yes

Describe style if “other”

Length_20 mins

0: No

1: Yes

Length_30 mins

0: No

1: Yes

Length_45 mins

0: No

1: Yes

Length_60 mins

0: No

1: Yes

Length_75 mins

0: No

1: Yes

Length_Other

0: No

1: Yes

Describe other

Pay per Bundle:

# of classes per $ amount

Karma Donation/Exchange

0: No, you have to pay using money

1: Yes, you can attend classes for free or reduced price if you can’t pay

2: Yes, you can donate your time/labor in exchange for classes

Locked Before Class

0: No, the door is not locked before class

1: Yes, the door is locked before/when class starts

999: No mention of door locking

Newsletter:

0: No, they have no newsletter

1: Yes, they have a newsletter/listerv

Blog:

0: No, they have no blog

1: Yes, they have a blog online accessible to anyone

Blog Last Updated:

Year of last update for blog

Facebook_Account

0: No

1: Yes

Facebook_Number of Followers

Twitter Account

0: No

1: Yes

Twitter Number of Followers

Instagram account

0: No

1: Yes

Instagram Number of Followers

Competitions/Prizes:

0: There are no competitions or prizes

1: Yes, there are competitions or prizes

Total instructors listed on website

200RYT Certified

Number of 200RYT certified instructors, use 999 if there are no instructors listed at all

500RYT Certified

Number of 500RYT certified instructors, use 999 if there are no instructors listed at all

Degree/other certifications

0: No instructor has any certifications other than RYT

1: Yes, *at least one* instructor has a degree or certification other than RYT listed

999: No instructors listed

Describe other certifications

Exercise for Health:

0: No mention of health benefits from exercise

1: Yes, they mention health benefits

Describe the exercise for health language

Nutrition:

0: No mention of nutrition

1: Yes, they mention nutrition in any form

Describe the nutrition language they use – supplement, detox tea, healthy diet, etc.

Meditation:

0: No mention of benefits of meditation

1: Yes, they mention meditation as being beneficial

Describe what they state the benefits of meditation are

Offer Teacher Training:

0: No they do not offer teacher training

1: Yes, they offer teacher training

Describe what training they offer

Appendix 2:Yoga Studio Coding Companion Sheet - Further Explanations/Definitions

| **Yoga Studio or Gym Name** | Self-explanatory |
| --- | --- |
| **City** | Self-explanatory |
| **Zip Code** | Self-explanatory |
| **Year Opened** | Self-explanatory |
| **Studio Capacity** | Number of students allowed per class |
| **# of yoga rooms** | Advertised number of rooms utilized for yoga.  Example: a hot yoga room and separate studio; “studio A” and “studio B” mentioned on class schedule or other pages |
| **Studio Type (gym vs boutique)** | *Gym*: facility that has yoga offered along with weights, cardio equipment, is open outside of class time, etc. Example: YMCA, university wellness centers  *Boutique*: facility that offers only classes, yoga or other group fitness classes, is only open during class times. Example: In Balance Yoga |
| **Studio Style** | Do they mention on the website a specific style/theme for the whole studio such as ‘hot yoga studio’, Bikram studio, meditation studio, etc. |
| **Registered with Yoga Alliance** | Are they registered with yoga alliance? Are they additionally registered/affliated with any other group? |
| **Studio Awards/Certifications** | Does the studio have any other awards or certifications? For example, a Best Small Business in the city, Best Yoga Studio in the city, etc. |
| **COVID-19 Classes** | Are offering COVID-19 classes online? |
| **COVID-19 Message** | Is there a statement/message about COVID-19 on the site? |
| **Diversity- Race/Ethnicity** | Photos on their website display a variety of ethnicities |
| **Diversity- Age** | Photos on their website display a variety of ages from young adults to seniors |
| **Diversity- Body Type** | Photos on their website display a variety of body types from small to large |
| **Diversity- Body Ability** | Photos on their website display participants using props (blocks, straps, extra mats, chairs) or prosthetic limbs, etc |
| **Diversity- Gender** | Photos on their website display a variety of genders |
| **Early Morning Classes** | Classes scheduled from 5:00am- 8:00am |
| **Morning Classes** | Classes scheduled from 8:01am-12:00pm |
| **Afternoon Classes** | Classes scheduled from 12:01pm-4:00pm |
| **Evening Classes** | Classes scheduled from 4:01pm-7:00pm |
| **Night Classes** | Classes scheduled from 7:01pm-midnight |
| **Total number of studio reported classes** | Does the studio state a number of classes they have available per week? |
| **Total number of reviewer calculated classes** | Count the number of classes available in the first 7 days of the month. |
| **Private Classes** | Private/individual classes can be set-up through the studio. |
| **Private class cost** | Cost in dollar per hour for a private class |
| **Type of schedule- online, have to download, not available** | Online: Schedule is available embedded into the website  Download: Schedule is in .pdf or other format that you click a link and it downloads onto your computer  Not available: No schedule on website/or ‘call for schedule’ |
| **Type of classes- Yoga Style** | At least one class available in each of the styles as stated on the schedule of classes or somewhere on the website. This is based on what the studio labels the class as, not reviewer interpretation. If they do not use one of the key words, code it under ‘other’ and describe. |
| **Temperature Reported** | Is studio temperature reported anywhere on the website? |
| **Room Temp 62-68** | At least one class listed as in a temperature range of 62-68 degrees |
| **Room Temp 68.1-88** | At least one class listed as in a temperature range of 68.1-88 degrees |
| **Room Temp 88.1- 100** | At least one class listed as in a temperature range of 88.1-100 degrees |
| **Room Temp >100** | At least one class listed as in a temperature range >100 degrees |
| **Length of Class** | Length in Minutes |
| **Cost per Class** | If available to pay on a class by class basis, cost in dollars per single class |
| **Cost per Month** | If available to pay for a month of classes, cost in dollars per month |
| **Pay per Bundle** | Yes or no, if they have bundle options (attend 5 classes, 6^th^ one is free, etc.) |
| **Pay at door** | Can you pay at the time of the class, or do you have to purchase a pass/membership online |
| **Cash or Card** | Can you pay by cash, gift card, or credit card |
| **Newsletter** | Do they have a newsletter for members/people on their listserv |
| **Social Media Account** | Do they have a twitter, tumblr, facebook, snapchat, instagram, tiktok or other social media account? |
| **Social Media Followers** | Add up all the followers the studio has across all social media accounts that the studio utilizes. |
| **Competitions/Prizes** | Are there prizes for attending the most classes, competitions through apps or their website for tracking workouts, etc |
| **Number of Instructors** | How many instructors are listed on the website |
| **200RYT Certified** | How many instructors listed on the website are 200RYT certified |
| **500RYT Certified** | How many instructors listed on the website are 500RYT certified |
| **Degree/other certifications** | Do any instructors have listed other relevant degrees or certifications, for example, a BS in exercise physiology or an AFAA group fitness certification |
| **Exercise for Health** | Does the website mention the benefits of exercise for health? Examples: ‘come to class to feel healthier’ ‘yoga is part of a healthy lifestyle’ |
| **Nutrition** | Does the website mention any nutritional recommendations or push any nutrition products? (evidence based products or detox teas, etc) |
| **Meditation** | Does the website mention meditation as an avenue for a better life/health? Examples: ‘people who meditate deal with stress better’ ‘people who practice mindfulness sleep better’ |

Appendix 3: Qualitative Themes Resulting from Analysis of Semi-Structured Interviews

| **Studio** | **Studio Open Date** | **Studio Goal** | **Website Reflection** | **Social Media Recruitment** | **Popularity Tracking** | **What Makes a class Popular** | **Most Popular Class Time** |
| --- | --- | --- | --- | --- | --- | --- | --- |
| **Breathing Space** | 1999 | End Suffering | Pleased | None | No  Tracking | -- | Morning/  Evening |
| **Just Breathe** | 2008 | Spread yoga to new people | Needs Tweaks | -- | No  Tracking | Individ-ualized  Attention | Morning |
| **Studio 221** | 2017 | Spread yoga to new people | Needs  Tweaks | Facebook,  Instagram | Attendance | Teacher and  Timing | Morning |
| **Vita-Zen** | 2010 | Spread yoga to new people | Pleased | Facebook,  Instagram,  Twitter | Attendance | Teacher, Class Type | Afternoon |
| **Uttarra** | -- | Spread yoga to new people | Pleased | Facebook,  Instagram | Attendance | Time,  Teacher | Morning |
| **Yoga Circle Downtown** | 1999 | Spread yoga to new people | Needs Tweaks | Not important | Attendance | Time, Teacher | Evening |
|  | **What Class Style is Most Popular** | **How do you make attendees feel welcome** | **Diversity-Staff** | **Diversity-**  **Students** | **8 Limbs and/or Sanskrit** | **Minimum Teacher Qualification** |  |
| **Just Breathe** | -- | Relationship | Age | -- | Both | -- |  |
| **Studio 221** | Iyengar | Relationship | -- | age | Yes | 200 RYT |  |
| **Vita-Zen** | Adaptive  Gentle | Relationship | None | Body Ability | Only Sanskrit | 200 RYT |  |
| **Uttarra** | Ageless Yoga | Relationship | None | Body Size, age, race | Both | 200 RYT |  |
| **Yoga Circle Downtown** | Relationship | Diverse | Diverse | Both | Unsure, No minimum | -- |  |
| **Breathing Space** | Relationship | Age, Ethnicity, Gender | -- | Both | Experience/hard certification | -- |  |
